# Opposing Fat Metabolism Pathways Triggered by a Single Gene

**DOI:** 10.1371/journal.pbio.0030083

**Published:** 2005-02-08

Regulating metabolism of fat is an important challenge for any animal, from nematodes to humans. Central players in the regulatory network are the nuclear hormone receptors (NHRs), which are transcription factors that turn on or off a set of target genes when bound by specific lipid molecules. NHR genes number 48 in mammals, and a surprising 248 in nematodes. Despite the difference in quantity, there are some structural similarities between NHRs in these two groups, in particular, between the nematode gene *nhr-49* and the mammalian *HNF4*. In this issue, Keith Yamamoto and colleagues show that *nhr-49* controls two different aspects of fat metabolism, which interact to form a feedback system controlling the consumption and composition of fats in the nematode.[Fig pbio-0030083-g001]

**Figure pbio-0030083-g001:**
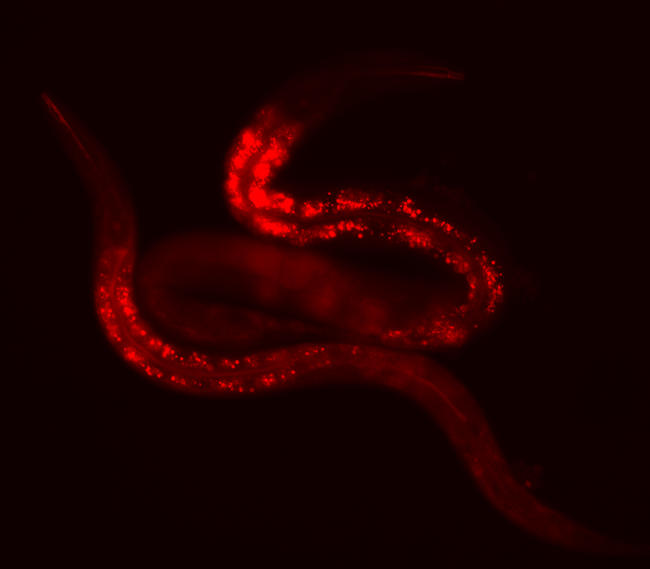
Regulation of fat content and lifespan in C. elegans

Using RNAi to suppress gene expression, the researchers discovered that when *nhr-49* was absent, the lifespan of the nematode was reduced by more than 50%, and the animal displayed numerous gross abnormalities in the gut and gonad. This was accompanied by unusually high fat content in the larvae. By using quantitative PCR to measure output of fat and glucose metabolism genes, the researchers showed that deletion of *nhr-49* changed expression of 13 of these genes, with the most dramatic effects occurring within two metabolic pathways: mitochondrial lipid oxidation and fatty acid desaturation.

Oxidation degrades lipids to release energy, explaining the build-up of fat in *nhr-49*-suppressed larvae. One of *nhr-49*'s normal functions is to increase expression of the mitochondrial acyl–Coenzyme A (CoA) synthetase gene *acs-2*. A principal role for mitochondrial acyl-CoA synthetases is to “activate” free fatty acids for transport into mitochondria, where they are oxidized. This process involves attaching a CoA group to a free fatty acid, and often serves as a rate-limiting step in lipid oxidation. Indeed, the authors found that suppression of *acs-2* alone was sufficient to reproduce the highfat phenotype, while overexpression of *acs-2* rescued the phenotype even in the absence of *nhr-49*.

Fatty acid desaturation is the process of converting saturated fats into unsaturated ones, by forming one or more double bonds between adjacent carbons in the tail. This process is catalyzed by fatty acid desaturase enzymes. *nhr-49* increases expression of several desaturases, most importantly *fat-7*, which converts stearic acid to oleic acid; deletion of *nhr-49* more than doubled the proportion of stearic acid compared to oleic acid.

RNAi interference of *fat-7* alone produced two interesting results. First, it shortened the nematode life span, suggesting this was the primary pathway through which *nhr-49* suppression exerted that same effect. Second, it produced some effects that were opposite those of *nhr-49* suppression: specifically, it reduced rather than increased fat content, and it increased rather than reduced expression of *acs-2*.

These results show that in its normal actions, *nhr-49* sets in motion two opposing pathways: it increases *acs-2*, which leads to reduction of fat content, and it increases *fat-7*, which, by reducing *acs-2*, increases fat content. Surprisingly, this behavior links *nhr-49* most closely not to *HNF4*, with which it shares the most structural similarity, but to another type of mammalian NHR, called peroxisome proliferator-activated receptors (PPARs). Further investigation of this link may lead to better understanding of the functions of PPARs, and provide opportunities for altering their function for treatment of fat metabolism disorders such as diabetes and obesity.

